# Melanoma Unknown Primary Brain Metastasis Treatment with ECHO-7 Oncolytic Virus Rigvir: A Case Report

**DOI:** 10.3389/fonc.2018.00043

**Published:** 2018-02-26

**Authors:** Guna Proboka, Andra Tilgase, Sergejs Isajevs, Agnija Rasa, Pēteris Alberts

**Affiliations:** ^1^Latvian Oncology Centre, Riga Eastern Clinical University Hospital, Riga, Latvia; ^2^R&D, International Virotherapy Center, Riga, Latvia; ^3^Department of Pathology, Riga Eastern Clinical University Hospital, Riga, Latvia; ^4^Department of Pathology, Faculty of Medicine, University of Latvia, Riga, Latvia

**Keywords:** melanoma brain metastasis, melanoma unknown primary, blood–brain barrier, oncolytic virus, ECHO-7 virus, intranasal, Rigvir^®^

## Abstract

Melanoma is considered an aggressive malignancy with a tendency of forming metastasis in the brain. Less than 10% of all melanoma cases present with unknown primary tumor location. This diagnose is yet to be fully understood, because there are only theoretical assumptions about the nature of the disease. Melanoma brain metastases have many severe side effects and, unfortunately, any disease related to the brain has limited therapeutic options due to the blood–brain barrier. The course of the disease after a treatment course is complicated to predict, and it is difficult to obtain long-lasting remission. In this report, we describe a female patient with unknown primary melanoma brain metastasis treated with the oncolytic ECHO-7 virus Rigvir^®^ after brain surgery. The patient has been stable, as monitored by magnetic resonance imaging, for more than 3.8 years with ongoing therapy. The median expected overall survival from the time of diagnosis is approximately 5 months. Additional positive effect could have been gained from use of the intranasal administration route, which is considered effective due to the direct anatomical connection between the nasal cavity and the central nervous system. However, further studies are required to fully understand this mode of drug administration.

## Introduction

A female patient born in 1954 has been reported to have had solar urticaria, chronic cholecystitis, hepatitis A virus infection twice, and frequent tonsillitis. In 2009, the patient had a basal cell carcinoma cutis abdominis removed; however, no surgical specimen or histological/pathological examination is available.

**Figure 1:**
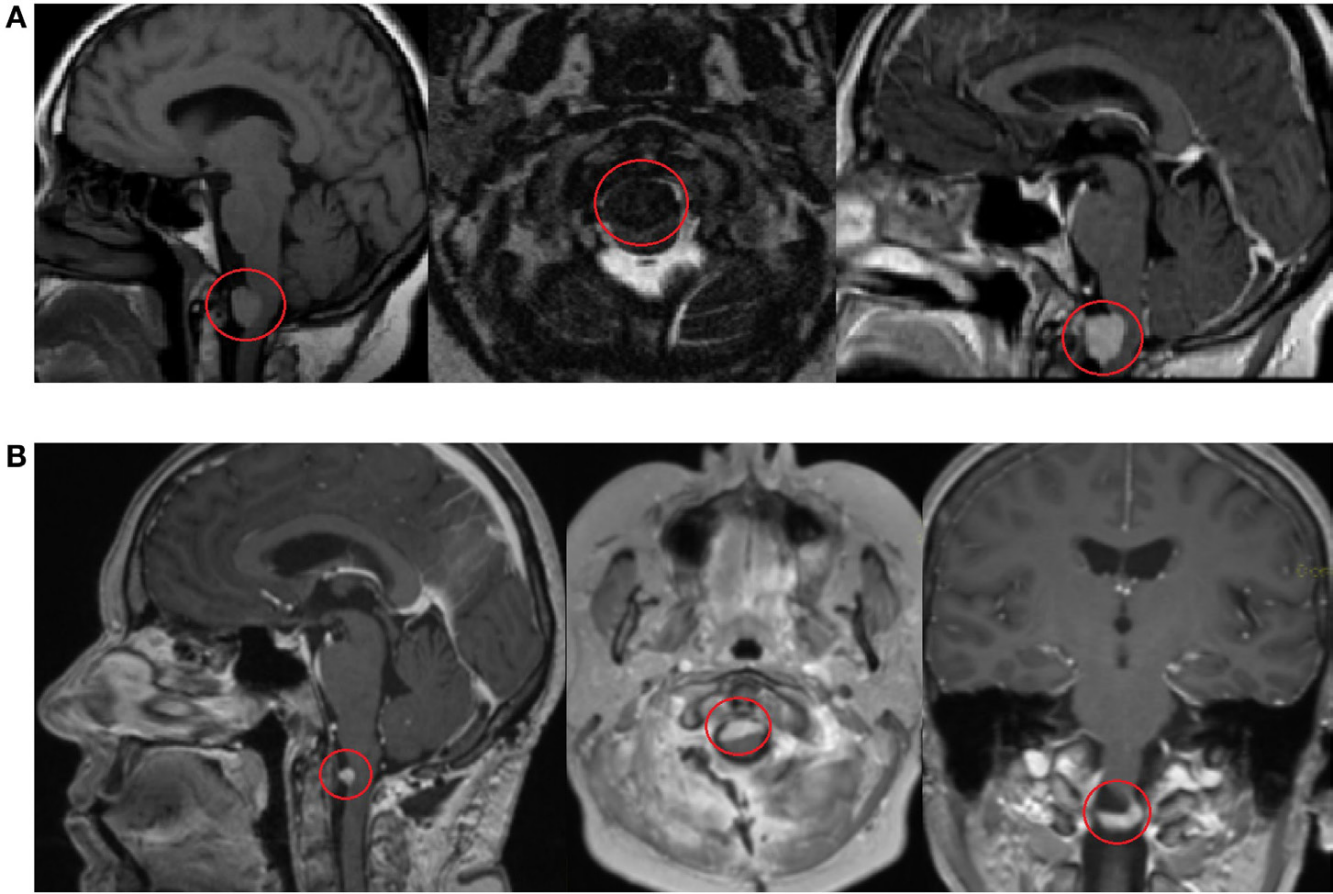
Contrast-enhanced head and brain magnetic resonance imaging (MRI) scan shows a formation in the craniospinal junction before surgery on 17 March 2014 **(A)**. Contrast-enhanced MRI scan 3 months after surgery shows a minimal, residual caudal part of the tumor on 5 June 2014 **(B)**.

In 2014, the patient complained about severe dizziness after movements and increased fatigue. No headache was observed. The patient has a Ph.D., she is married, has three children, her hobbies include traveling and has an active lifestyle. There is no previous family history of cancer. After contrast-enhanced head and brain magnetic resonance imaging (MRI), the patient was diagnosed with a formation in the craniospinal junction. The patient underwent a planned posterolateral foramen magnum formation extirpation in 23 April 2014 (Figure 1). The surgery lasted for 12 h, and no severe complication was observed. CT scan performed 5 days after surgery showed a minimal residual caudal part of the tumor. The condition of the patient during the postoperative period was satisfactory; progressive renewal of physical endurance with no signs of new essential neurologic deficit was observed.

In order to localize the primary tumor, the patient has undergone several examinations: CT scans of the lungs and abdomen, colonoscopy, endoscopy (not shown), and consultations with a dermatologist and an ophthalmologist. The melanoma primary tumor has not been found.

**Figure 2:**
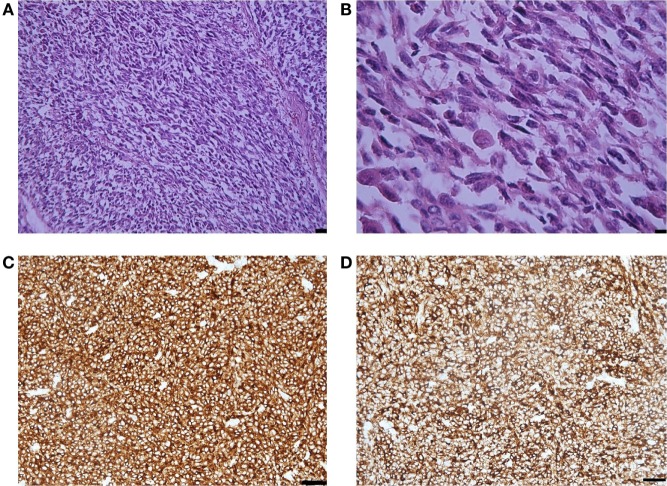
**(A)** Representative photomicrographs of the melanoma metastasis: epithelioid and spindle cells. Hematoxylin-eosin staining, magnification ×40, scale bar is 50 µm. **(B)** The tumor cells demonstrated marked nuclear pleomorphism with variation in cell size, shape, and staining and increased mitotic activity. Hematoxylin-eosin staining, magnification ×200, scale bar is 25 µm. **(C)** MART-1 immunopositivity in the tumor cells. Immunohistochemical staining method, magnification ×200, scale bar is 25 µm. **(D)** S-100 immunopositivity in the tumor cells. Immunohistochemical staining method, magnification ×200, scale bar is 25 µm.

Histological examination showed that the metastasis was composed of spindle and epithelioid cells, with large round and elongated nuclei with prominent vesicular nuclei and large nucleoli and abundant eosinophilic cytoplasm, surrounded by mature collagen bundles. The tumor cells demonstrated marked nuclear pleomorphism with variation in cell size, shape, and staining. Mitotic activity was up to five mitotic figures per high powered field (at magnification ×200). Melanin pigment was not observed by hematoxylin eosin staining (Figure 2). Immunohistochemistry demonstrated that the tumor cells were weakly positive for HMB-45 and Melan A, strongly positive for MART-1, S-100, and vimentin, and that the Ki-67 index was 35% (Figure 2). BRAF gene mutations in codons *V600E, V600K, and V600D* were not detected.

The patient declined postoperative radiation therapy, because the metastasis residue was closely located to truncus encephali and; therefore, the risk of complications was estimated as high, and efficacy of the radiotherapy uncertain (at that time, stereotactic radiosurgery was not available in Latvia). Since no BRAF mutation was found, therapy with a BRAF inhibitor was contraindicated; in 2014, CTLA-4 and PD-1 immunotherapy was not registered in Latvia (at the time when the decision on adjuvant therapy was made).

An apparently successful long-term Rigvir treatment of a stage IV M1c melanoma patient has previously been reported. Rigvir monotherapy was prescribed after the patient had developed severe side effects from chemotherapy (1).

Therefore, virotherapy with Rigvir^®^ was chosen. The therapy was started in July 2014 with three intramuscular administrations of 2 ml for three consecutive days with the third divided into 1.5 ml intramuscular and 0.5 ml intranasal administration. Subsequently, administrations were once per week, the first two intramuscular and the third divided into intramuscular and intranasal administration. From January 2015, the administrations were changed to one injection every 2 weeks, and from December 2016, to one every 3 weeks, with every third divided into intramuscular and intranasal administration. The patient has not received any other cancer treatment pre- or postsurgery. Serum clinical chemistry parameters were recorded and graded according to NCI CTCAE. Values above grade 1 were not observed during Rigvir^®^ therapy.

**Figure 3:**
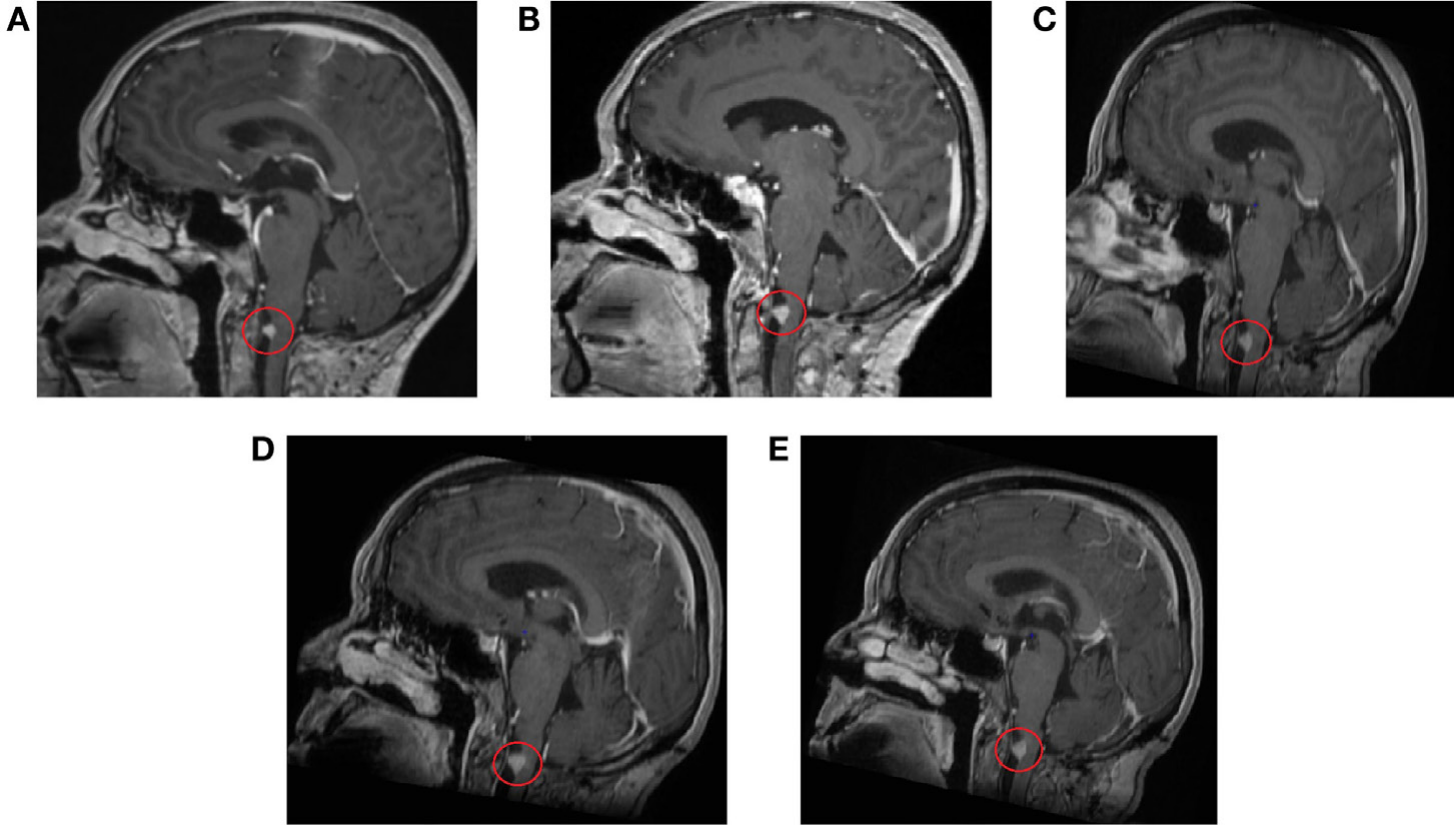
Contrast-enhanced magnetic resonance imaging scans of the head and brain show residual tissue of the melanoma metastasis without significant changes in structure and size. **(A)** 11 September 2014, **(B)** 5 March 2015, **(C)** 29 September 2015, **(D)** 29 February 2016, **(E)** 2 August 2016.

Several follow-up MRI scans of the head and brain have been made (5 June 2014 to 2 August 2016) (Figure 3). All scans show minimal residual tissue of a melanoma metastasis in the anterior spinal cord located in the craniospinal junction without significant changes in structure and size. Signs of central and cortical atrophy (September 2015) and periventricular and subcortical vascular leukoencephalopathy with distinct and converging foci of vascular genesis in cerebral hemispheres were observed. Comparison of the last two scans yields that no change in the focus was observed.

## Background

Melanoma is an aggressive malignancy with an increasing incidence worldwide. It has been noted that malignant melanoma has a high risk for dissemination, both through lymphatic and hematogenous pathways (2).

Usually, the primary tumor of melanoma can be detected on the skin, mucosa, or ocular tissue and is classified as melanoma known primary. However, melanoma may also present without known primary tumor, called melanoma unknown primary. It is estimated that 2–6% of all melanoma patients have unknown primary tumor (3). The pathogenesis of melanoma unknown primary is still not fully understood. Hypothetically, unknown primary melanoma is suggested to arise from primary melanoma that has spontaneously regressed and cannot be detected (4). Other hypotheses suggest that ectopic nevus cells residing in visceral organs, lymph nodes, or the nervous system may also give rise to malignant melanoma. Finally, the patient may have had the formation removed without histological diagnosis or the formation might have been misdiagnosed (3). In cases of recurrence, re-biopsy has been described as a successful approach to suggest the origin of cancer and elucidate possible therapeutic approaches (5).

The brain is a favorable environment for the development of metastases due to processes like migration, proliferation, and angiogenesis (6, 7). Melanoma brain metastases are associated with complications like hemorrhage, increased intracranial pressure, and focal or generalized seizures. In such cases, it is recognized that it is difficult to attain long-lasting remission after the cancer treatment is stopped. To achieve a favorable disease course, it is necessary to consider multimodality strategies (6–8).

Therapies for melanoma brain metastasis are limited mainly because the blood–brain barrier limits the access of circulating molecules from entering the brain parenchyma; therefore, the use of traditional cytotoxic chemotherapy is limited. The usually prescribed treatments for brain metastases are surgery and/or radiation therapy. For patients with advanced-stage melanoma, BRAF-targeted regimens and immunotherapy have shown some efficacy improving the overall survival. It has been suggested that BRAF-targeted regimens might show efficacy in patients with brain metastases (6, 8).

## Discussion

The present patient had a basal cell carcinoma excised 5 years prior to the brain metastasis surgery. However, there is no histological record available confirming the basal cell carcinoma diagnosis. In 2014, a melanoma brain metastasis was identified but no primary tumor was found. It is not clear if the basal cell carcinoma might have been misdiagnosed. In contrast, histological examination of the material obtained at the brain surgery has been performed using the appropriate melanoma markers (HMB-45 and Melan A weakly positive, MART-1, S-100, and vimentin strongly positive).

Treatment options for brain metastasis are limited due to the blood–brain barrier. However, picornaviruses, to whom the oncolytic ECHO-7 virus Rigvir^®^ belongs, are known to be able to infect the central nervous system (9, 10). The blood–brain barrier consists of polarized microvascular endothelial cells that restrict access to the central nervous system by forming tight junctions. These junctions confine the flow to macromolecules, ions, and solutes. Several routes have been reported by which viruses can cross the blood–brain barrier: by direct infection of endothelium, by crossing through the paracellular space, and by infecting immune cells that can migrate through the epithelium. Several studies have investigated the possible use of intranasal administration of oncolytic viruses by the stem cells to treat glioblastoma. The intranasal administration route is considered to be one of the most beneficial due to several reasons. First, there is a direct connection between the nasal cavity and the central nervous system, second, it is the only possible mode of administration that is not invasive. Third, it is possible to overcome the blood–brain barrier (11). However, most of these studies were done on rodents, and the difference between human and rodent brain must be taken into consideration. Due to possible benefits of this mode of administration, further research is necessary (12–14).

The median expected overall survival from the time of melanoma brain metastasis diagnosis is approximately 5 months (15). A meta-analysis suggests that the prognosis of melanoma unknown primary is better than that of melanoma known primary (4). The patient described here has been stable for more than 3 years and 9 months, and the virotherapy is still ongoing.

## Concluding Remarks

It has been previously suggested that Rigvir^®^ improves the overall survival in patients with stage IB–IIC melanoma (16). The present report describes a melanoma unknown primary brain metastasis patient treated with Rigvir^®^ virotherapy. There are limited options to treat brain metastasis because of the brain–blood barrier. The present patient has been stabilized following Rigvir^®^ virotherapy, suggesting that the oncolytic virus Rigvir^®^ might have reached the brain metastasis. Further studies are necessary to elucidate the possible mechanisms of action.

## Patient Consent Statement

Written consent to publication was obtained from the patient.

## Author Contributions

GP is the attending immunologist, oncologist of the patient, and SI carried out the histology and pathology analysis. AT, AR, and PA made substantial contributions to acquisition, analysis, and interpretation of data and drafted the manuscript. All authors have read and approved the final version of the manuscript for publication.

## Conflict of Interest Statement

GP, PA, AT, and AR are employees of the International Virotherapy Center. SI have no potential conflicts of interest to disclose.
