# Erratum: Melanoma Unknown Primary Brain Metastasis Treatment With ECHO-7 Oncolytic Virus Rigvir: A Case Report

**DOI:** 10.3389/fonc.2018.00172

**Published:** 2018-05-18

**Authors:** Frontiers Production Office

**Affiliations:** Frontiers Media SA, Lausanne, Switzerland

**Keywords:** melanoma brain metastasis, melanoma unknown primary, blood–brain barrier, oncolytic virus, ECHO-7 virus, intranasal, Rigvir^®^

In the original article, we neglected to include the patient consent statement.

## Patient Consent Statement

Written consent to publication was obtained from the patient.

The Production office apologize for this error and state that this does not change the scientific conclusions of the article in any way.

The original article was updated.

## Conflict of Interest Statement

The authors declare that the research was conducted in the absence of any commercial or financial relationships that could be construed as a potential conflict of interest.

